# MeT-DB: a database of transcriptome methylation in mammalian cells

**DOI:** 10.1093/nar/gku1024

**Published:** 2014-11-06

**Authors:** Hui Liu, Mario A Flores, Jia Meng, Lin Zhang, Xinyu Zhao, Manjeet K. Rao, Yidong Chen, Yufei Huang

**Affiliations:** 1 School of Information and Electrical Engineering, China University of Mining and Technology, Xuzhou, Jiangsu 221116, China; 2 Department of Electrical and Computer Engineering, University of Texas at San Antonio, TX 78249–0669, USA; 3 Department of Biological Sciences, Xi'an Jiaotong-Liverpool University, Suzhou, Jiangsu 215123, China; 4 Greehey Children's Cancer Research Institute, University of Texas Health Science Center at San Antonio, TX 78229, USA; 5 Department of Cellular Structural Biology, University of Texas Health Science Center at San Antonio, TX 78229, USA; 6 Department of Epidemiology and Biostatistics, University of Texas Health Science Center at San Antonio, TX 78229, USA

## Abstract

Methyltranscriptome is an exciting new area that studies the mechanisms and functions of methylation in transcripts. The MethylTranscriptome DataBase (MeT-DB, http://compgenomics.utsa.edu/methylation/) is the first comprehensive resource for N6-methyladenosine (m^6^A) in mammalian transcriptome. It includes a database that records publicaly available data sets from methylated RNA immunoprecipitation sequencing (MeRIP-Seq), a recently developed technology for interrogating m^6^A methyltranscriptome. MeT-DB includes ∼300k m^6^A methylation sites in 74 MeRIP-Seq samples from 22 different experimental conditions predicted by exomePeak and MACS2 algorithms. To explore this rich information, MeT-DB also provides a genome browser to query and visualize context-specific m^6^A methylation under different conditions. MeT-DB also includes the binding site data of microRNA, splicing factor and RNA binding proteins in the browser window for comparison with m^6^A sites and for exploring the potential functions of m^6^A. Analysis of differential m^6^A methylation and the related differential gene expression under two conditions is also available in the browser. A global perspective of the genome-wide distribution of m^6^A methylation in all the data is provided in circular ideograms, which also act as a navigation portal. The query results and the entire data set can be exported to assist publication and additional analysis.

## INTRODUCTION

The past decades have witnessed the great progress in DNA methylation research (1,2). Yet, research on methylation in transcripts is still at its very early stage. Thus far, N6-methyl-adenosine (m^6^A) is known to be the most abound form of methylation in transcripts. The recent surge of interest in transcriptome-wide m^6^A methylation stemmed largely from two seminal studies (3,4), where a new enrichment-based high-throughput method called MeRIP-Seq, or methylated RNA immunoprecipitation sequencing, was introduced to identify for the first time the presence of over 12 000 m^6^A sites in about 20% of mammalian genes. Transcriptome-wide analysis also revealed that m^6^A sites reside in the highly evolutionarily conserved regions with a consensus sequence motif of RRACH that is also conserved between human and mouse, suggesting the possibility of conserved regulatory functions of m^6^A. A series of subsequent papers that used MeRIP-Seq and other high-throughput methods have started to produce evidences and new hypotheses on involvement of m^6^A in regulating many functions (5).

Currently, analysis of m^6^A distribution in mRNA showed that m^6^A is enriched in the 3′ untranslated region (UTR) and near stop codon. Among 3′UTRs that contain an m^6^A site, two-thirds also have at least one TargetScan-predicted miRNA binding site, suggesting that m^6^A might be involved in miRNA-mediated gene silencing. Existing studies also suggested that m^6^A recruits different RNA binding proteins (RBP) to mediate its own methylation cycle and to regulate various other cellular functions. For instance, transcriptome m^6^A methylation is known to be catalyzed by the methyltransferase complex that includes methyltransferase-like 3 (METTL3), Wilms tumor 1-associated protein (WTAP) and METTL14 (6). Interestingly, WTAP is a pre-mRNA-splicing regulator and a recent PAR-CLIP analysis also showed that METTL3 binding sites reside mostly in intronic regions of pre-mRNA (7). These evidences suggest a likely involvement of m^6^A in splicing but the splicing factors and the mechanism by which it regulates splicing is unclear. In addition to splicing, m^6^A has also been shown to promote mRNA degradation through recruiting the human YTH domain family 2 protein to the methylated mRNA and this recruitment redirects mRNA to mRNA decay compartment as opposed to ribosome for translation (8). In contrast, m^6^A was also suggested to stabilize mRNA, where loss of m^6^A in an mRNA is coupled with decrease in its expression level. The degree and context by which m^6^A would choose to meditate mRNA degradation over stability is completely unknown. Recent studies also showed that m^6^A methylation can be reversed by two endogenous enzymes, obesity-associated protein (FTO) and α-ketoglutarate-dependent dioxygenase alkB homolog 5 (ALKBH5) (9). However, the functional differences between the two demethylases and whether there exists additional demethylase are still elusive. Interestingly, knockout FTO in mouse midbrain impairs dopamine dopaminergic activity, which suggested that m^6^A might indirectly control neuronal activity and behavioral responses. These exciting advancements in transcriptome m^6^A methylation have inspired a new research area now known as methyltranscriptome. Continuing effort in deciphering m^6^A functions under various contexts represents a grand challenge to the biology community in coming years (10).

Along with the increased amount of m^6^A MeRIP-Seq data sets, there is a strong need for an integrated database that facilitates the exploration of these data sets. To meet this need, we created the first integrated MethylTranscriptome DataBase or MeT-DB (http://compgenomics.utsa.edu/methylation/). MeT-DB includes a comprehensive collection of m^6^A sites predicted from all published MeRIP-Seq data sets. To enhance its utility, MeT-DB also integrated m^6^A sites with the binding sites of miRNA, splicing factors and several RBP. It also includes gene expression data for every MeRIP-Seq data samples. The web-based interface also includes an internal genome browser with browsing and query functionalities optimized for exploration and analyses of MeRIP-Seq data. Particularly, the browser provides the analysis of differential methylation and differential expression of m^6^A methylated genes under two conditions recorded in the database. MeT-DB will help shed lights on the biological functions of m^6^A methyltranscriptome.

## MATERIALS AND METHODS

**Figure 1.**
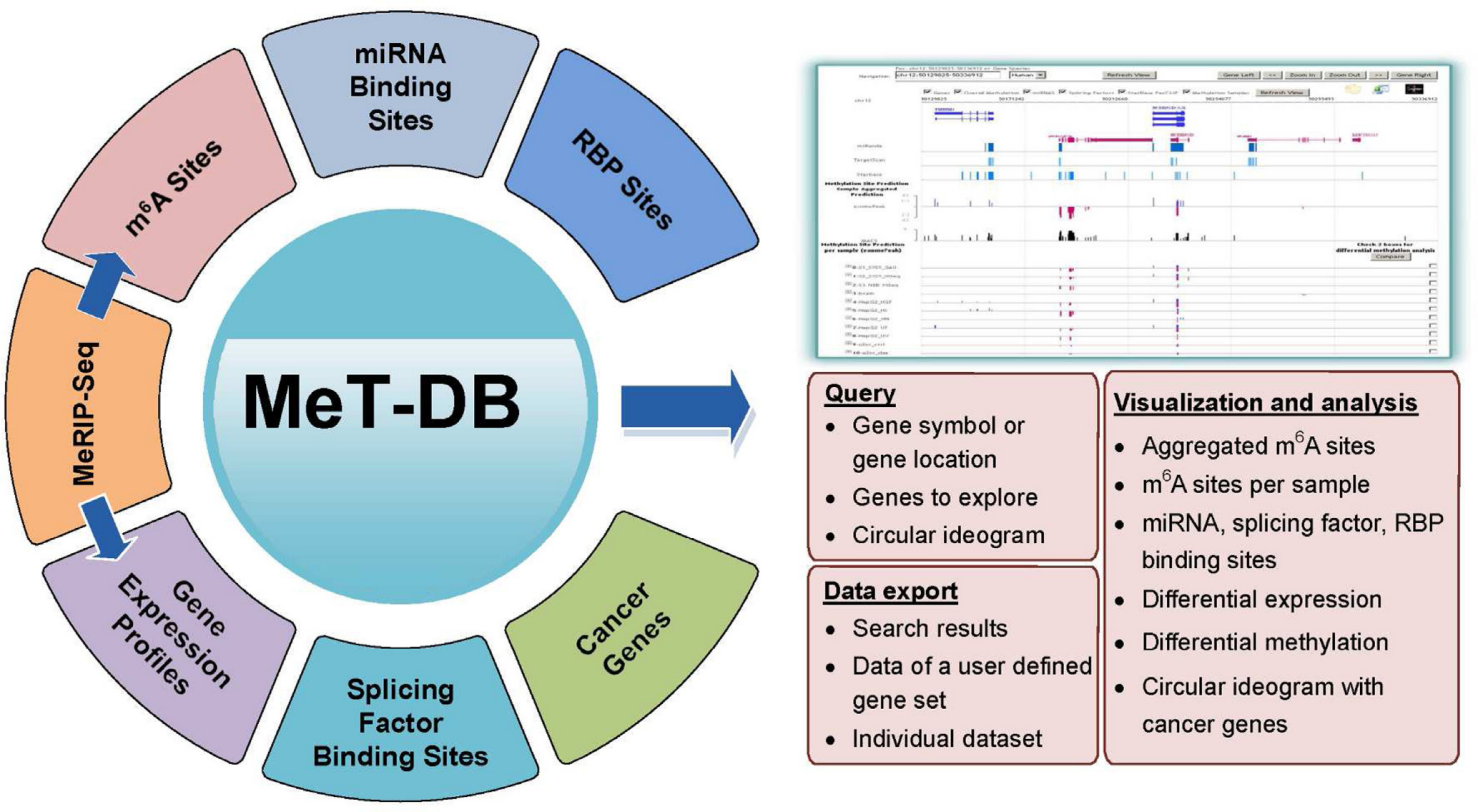
Overall design of MeT-DB database. MeT-DB includes all public available mammalian MeRIP-Seq data sets for RNA methylation sites predicted using exomePeak and MACS2 algorithms. Other related data including binding sites of miRNAs, splicing factors, and RNA binding proteins as well as gene expression profiles were also integrated. MeT-DB also includes an internal genome browser to facilitate data query, visualization, analysis and export.

A schematic diagram illustrating data sets and functionality of MeT-DB is shown in Figure 1. Currently, MeT-DB includes MeRIP-Seq and other related data for human (UCSC hg19) and mouse (UCSC mm9).

### MeRIP-Seq data

The MeRIP-Seq technology combines the essential technologies of methylated DNA immunoprecipitation (MeDIP), immunoprecipitation of RNA-binding proteins (RIP) and RNA sequencing (RNA-Seq) to enable high-resolution detection of transcriptome-wide methylation. MeRIP-Seq immunoprecipitates heavily fragmented, methylated transcripts [including mRNAs and long non-coding RNAs (lncRNAs)] and then sequences the pull-down RNA fragments for computational processing. Two types of samples, the immunoprecipitated (IP) and the control, are obtained for each experiment, where an IP sample includes sequenced reads from the methylated fragments that are immunoprecipitated and a control sample includes the sequenced reads from fragments in the cell transcriptome, which is essentially the RNA-Seq measurement of transcript abundance. The methylation sties, or peaks, are detected by examining the enrichment of the reads in IP samples relative to those in input samples (11).

MeT-DB includes a total of 74 m^6^A MeRIP-Seq IP/input samples (Supplementary Table S1) from 22 experimental conditions (GSE29714, GSE37005, GSE48037, GSE47217, GSE46705; Supplementary Table S2) collected from five independent studies (3,4,8,12,13) and the data sets were downloaded from NCBI Gene Expression Omnibus (GEO; http://www.ncbi.nlm.nih.gov/geo/) and/or Short Read Archive (SRA; http://www.ncbi.nlm.nih.gov/sra). The data set (GSE46880) from (14) was excluded because it has no input samples and thus does not conform completely to the MeRIP-Seq protocol. Methylation site prediction, or MeRIP-Seq peak calling, was performed on the data sets from each of the 22 conditions according to the following two processing pipelines. The first pipeline was proposed in (3), where for each condition, MeRIP-Seq IP and input sample files are first concatenated into a single IP and input fastq file, respectively. Then, reads in the IP/input files are aligned to the genome by Tophat2 (version 2.0.11) (15), which implicitly calls Bowtie2 (version 2.1.0) with default options. Afterwards, peak calling is performed on the input and IP BAM files by MACS2 (version 2.0.10.20131216) (16), a chromatin immunoprecipitation sequencing (ChIP-Seq) peak calling algorithm, with default significance level 0.05. The second pipeline is based on a specialized MeRIP-Seq peak calling tool, exomePeak R/Bioconductor package (11). exomePeak adopts an exome-based approach, which performs peak calling on the pooled exons of a gene instead of on the genome as MACS2 does to account for peaks spanning exon–exon junctions. As a result, exomePeak can also report the strand information of predicted peaks. More accurate background estimation is also implemented in exomePeak by using only reads from the exonic regions. exomePeak has been shown to achieve higher specificity than MACS2 in predicting methylation sites from MeRIP-Seq data (11). In this second pipeline, each IP/input sample is first aligned to the reference genome by Tophat2, and then exomePeak is applied to call peaks using all the samples from a condition with default options. The called peaks from biological replicates are merged internally by exomePeak package.

For each predicted RNA methylation site, its chromosomal location including start/end position, strand information (only for exomePeak result) and fold enrichment are reported. In addition, annotation information is generated, including the gene identifiers of transcript (UCSC ID, Entrez Gene ID, Gene Symbol, RefSeq ID) as well as the information about whether the m^6^A site is located on 5’UTR/Coding DNA Sequences (CDS)/3’UTR or mRNA/lncRNA, where a lncRNA is defined as a transcript with exonicregions longer than 200nt and without any known CDS defined in the transcript database.

### Gene expression

Genome-wide expression levels for each of the 22 conditions were estimated from MeRIP-Seq input samples. Reads were first aligned to the corresponding genome (hg19 or mm9) with Tophat2 (15), and then Cufflinks (v2.1.1) (17) was employed to estimate the gene expression profiles with default settings and by using RefSeq gene annotations (http://cufflinks.cbcb.umd.edu/igenomes.html).

### miRNA target sites

To facilitate exploration of m^6^A and miRNA regulation, predicted miRNA target sites from TargetScan (version 6.2) (18) and miRanda (August 2010 release) (19) as well as StarBase (version 2.0) (20) recorded sites that are detected by PAR-CLIP experiments were included.

### Splicing factor and RNA binding protein binding sites

Six-hundred-and-fifty-five and one-hundred-and-twenty-five binding sties of human and mouse splicing factors, respectively, were obtained from SpliceAidF (v1.1 03/2013) (21). Each site includes the name of the binding splicing factor and the genome location of the binding site. Similarly, PAR-CLIP and HITS-CLIP detected binding locations of 24 human and 5 mouse RBPs were retrieved from StarBase.

### Gene annotation and cancer genes

We use UCSC hg19 and mm9/refFlat table for transcript annotation, which includes RefSeq ID, symbols and other positional information. For human genes, we also downloaded HUGO annotation (August 2014) to extract all official gene symbols, as well as their associated ‘previous Name’ or ‘Synonyms’ to map all possible gene symbol input. m^6^A has also been implicated to be involved in cancer (22–24). To help further investigate this association, cancer genes and tumor suppressor genes were also imported to the database. Seven-hundred-and-sixty-one human and six-hundred-and-twenty-eight mouse tumor suppressor genes were obtained from TSGene database (25). In addition, 522 cancer genes were downloaded from COSMIC (v70) (26).

### Database implementation, circular ideogram, web interface and availability

All data sets were processed and stored in a MySQL Database Management System (version 5.1.69) installed on an X86–64 server with Redhat Linux GNU OS. The database consists of 101 tables that comprise ∼1.74 billion records.

Circular ideograms at both genome and chromosome levels were created to display the distribution of exomePeak predicted sites. To generate an ideogram image, the site location data were parsed and formatted into data of segments, where for the chromosome image, a segment is 1 million bp in length, whereas for the genome image, a segment includes 5 million bp. The formatted site location data were then passed, along with the formatted locations of cancer gene data, to Circos (27) for image generation, where the methylation fold enrichments were scaled logarithmically. At last, individual links to display the data in the browser were added to different sections and cancer genes in the ideogram.

A web application including an internal genome browser was designed to provide user-friendly access to database searching and data visualization. The database query, genome browser and user interface were developed using PHP 5.3.3, JavaScript, WZ Jsgraphics library v. 3.05, jQuery JavaScript library v1.8.3, XHTML 1.0 and SVG 1.1. The web application is publicly accessible at http://compgenomics.utsa.edu/methylation/ and the Frequent Asked Questions (FAQ) section of the website provides detailed description of its functions and features.

## RESULTS

### Predicted m^6^A sites

**Figure 2.**
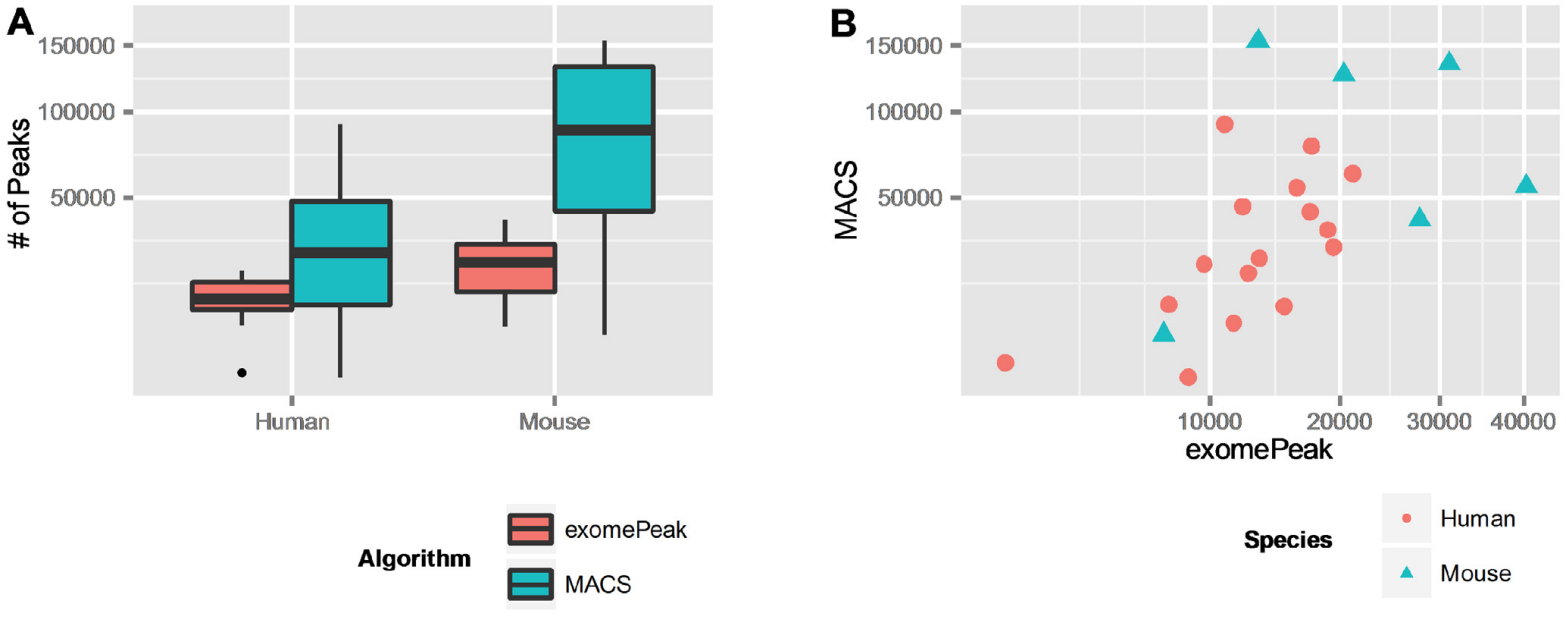
Number of m^6^A sites identified by MACS2 and exomePeak in human and mouse. (**A**) In general, for both human and mouse, MACS2 identified much more peaks than exomePeak. This is mostly because MACS2 reports a single peak that spans multiple exons as multiple peaks. (**B**) The number of peaks identified by MACS2 and exomePeak are highly correlated under the same condition.

A large number of m^6^A sites were identified by exomePeak and MACS2 from these 22 MeRIP-Seq data sets, and the result is summarized in Table 1 and Supplementary Table S3-S6. On average, MACS2 with the default setting detected more sites than exomePeak per condition (33 953 versus 13 345 for human and 86 245 versus 22 324 for mouse; Figure 2A). This is expected because MACS2 reports a single peak that spans multiple exons as multiple peaks. However, for the same data set, the numbers of peaks reported by exomePeak and MACS2 are significantly correlated (Pearson correlation 0.6743622; *P*-value = 0.0005777; Figure 2B).

**Table 1.** Total number of identified m^6^A sites

Species	Algorithm	Assembly	Total peak #	# of conditions	Average peak #
Human	exomePeak	hg19	213 530	16	13 345
Human	MACS2	hg19	543 088	16	33 943
Mouse	exomePeak	mm9	139 947	6	22 324
Mouse	MACS2	mm9	517 473	6	86 245

For complete result of individual condition, refer to Supplementary Tables S3–S6.

MEME suite (28) was also employed to predict the sequence motif of exomePeak detected m^6^A sites. Despite large variation in the numbers of detected m^6^A sites across different MeRIP-Seq conditions, the most enriched consensus sequences are all similar to the previously reported RRACH motif of m^6^A (3,4) (Supplementary Table S7), suggesting that the exomePeak detected m^6^A sites should have high specificity.

### Data browser

A web interface with an internal browser was developed for integrative exploration of the m^6^A sites predicted by exomePeak and MACS2 under different conditions (Supplemental Figure S1). The interface displays query results in a browser, which includes multiple tracks for visualizing the data. The gene tracks show the coordinates and structures of UCSC RefSeq genes. The gene symbols are displayed at the top of each gene track. The gene structure including UTRs, exons, introns and the strand direction is displayed according to the conventions of the UCSC genome browser. The strand information is also color-coded (blue for the positive strand and red for the negative strand) in addition to the arrows that indicate the direction from 5′ to 3′ of a gene. The site prediction tracks include the predicted sites and their degree of enrichment for each of the conditions as well as aggregated from all conditions. Each track displays bars spanning the predicted sites, whose heights are proportional to their methylation enrichments and whose color define their strand information consistent with that for gene tracks. The browser also includes tracks that display the miRNA target sites data from miRanda, TargetScan and StarBase, the splicing factor binding data from SpliceAidF, and StarBase CLIP data for RBP sites. Vertical lines are displayed at the location of all these sites and the name of the binding miRNA/splicing factor/RBP is shown when moving the cursor over the bar. In addition to tracks that display different data, the browser also provides a series of functions including navigation, track selection, data export, import data and linking to UCSC Genome Browser to display the present region of the genome. An alignment line that follows the cursor was also implemented to help align the same location in different tracks. The user can find more detailed description of the browser functions in the FAQ/Tutorial page of the website.

### Circular ideogram

The circular ideograms display exomePeak predicted m^6^A sites and the methylation enrichment at genome and chromosome levels (Supplementary Figure S2). The genome-level ideogram is clickable by chromosome, which will link to a chromosome ideogram, where the user can select a region within the chromosome or click a predefined cancer gene placed on the circular ideogram to display the associated data in the browser.

### Query the database

The database can be queried in three different ways. First, it is possible to search by ‘Gene Symbol’, ‘RefSeq ID’ or ‘Locus’ by entering this information in the search box appeared in the homepage as well as the ‘Browse’ page. Second, the user can query through the circular ideogram by selecting a region of interest or a cancer gene. Third, the user can also select from the set of pre-linked genes of interest in the ‘Genes to explore’ section of the homepage. Clicking on a gene will trigger the display of default tracks in the browser.

### Differential methylation and differential expression

The users can also investigate differential methylation as well as differential gene expression, from which the relationship between the context specific methylation and gene expression can be observed. To perform this analysis, the user can select the exomePeak/MACS2 predictions tracks from any two conditions by checking the checkboxes next to the two tracks. By clicking the ‘Compare’ button, the differential m^6^A methylation of the sites (methylation enrichment value from Condition 1 subtracts that of Condition 2) in the displayed region between the two selected tracks (the first checked checkbox counting from top will be considered as Condition 1 and the other is Condition 2 experiment) and the corresponding differential expression will be calculated (log2-transformed expression level of Condition 1 subtracted that of Condition 2) and displayed. For the differential methylation track, bars are placed at the differential methylation sites, where an up-right bar represents hyper-methylation (more methylation enrichment in condition one), a down-ward bar represents hypo-methylation (less methylation enrichment in condition one) and the height of the bar is proportion to the degree of differential methylation (absolute logarithm fold peak enrichment). For the differential expression track, a block that spans the entire region of a differential expressed gene is placed, whose color indicates the state of differential expression (up-regulation in red, down-regulation in green and no-change in black).

### Custom track data upload

User can upload one track of his/her own peak calling result in BED format (either common-delimited or tab-delimited. For example, chr17, 7559000, 7560000, 2). The input text-box is at the upper-right corner, next to UCSC logo. The custom track will be displayed below aggregated/consensus tracks, and only peaks within current viewing genomic regions will be displayed. The uploaded data will not be stored in the server, and user can remove the track by pressing the ‘Remove’ button below the input text-box.

### Data export

There are three different ways that the user can export the data. First, predicted sites by exomePeak and MACS2 from all tracks in the displayed genomic region of the browser can be exported by clicking the ‘Export Data’ icon at the upper-right corner of the browser. Second, peak prediction results of a user defined gene set can be exported as a common-separated value (CSV) file from the ‘Download’ page. Third, peak calling results for every sample by exomePeak or MACS2 can be downloaded from Table 3 in the ‘Download’ page.

### Utility of MeT-DB: a working example

If the user is interested in investigating any potential influence of m^6^A on a tumor suppressor gene, BRCA1, a query can be initiated by directly clicking the name BRCA1 listed in the ‘Genes to explore’ section of the homepage or by entering ‘BRCA1’ or its gene coordinate in the search box. The query results from the database including all the predicted m^6^A peaks in all BRCA1 isoforms by exomePeak and MACS2 as well as the binding sites of BRCA1 related miRNAs, splicing factors and RBPs are displayed in the genome browser (Supplementary Figure S1). The gene track shows that BRCA1 has six different isoforms. Examining the common peaks predicted by exomePeak and MACS2 from the aggregated prediction tracks revealed two clusters of m^6^A sites, one in the stop codon and 3’UTR and the other in an exon of four (4) BRCA1 isoforms. In the BRCA1 3’UTR, miRanda, TargetScan and StarBase all reported multiple target sites from a large number of miRNAs, some of which are inside the m^6^A sites. The user can further examine the tracks of splicing factor and RBP sites, which reveals a binding site of splicing factor Hu Antigen R in the vicinity of m^6^A sites in the 3’UTR. For the second m^6^A site cluster, the user might be interested in investigating the conditions that lead to this isoform specific m^6^A methylation by inspecting the ‘predictions per sample’ tracks. In this case, the m^6^A site cluster is predicted by both algorithms to exist in sample ‘HEK293T_S1’, ‘HEK293T_S1’, ‘HeLa2_ctrl’ and ‘HeLa2_KO_M3’, where the first two came from HEK293T cells, while the latter two came from HeLa cells. The user can also inspect the reads densities in the corresponding input and IP samples of ‘HEK293T_S1’ by turning on the plus icon at the left side of the sample label ‘HEK293T_S1’. Reads enrichment in the IP sample relative to the input sample is visible at both predicted site clusters. If the user wishes to further assess the differential m^6^A methylation and the differential gene expression between sample ‘HEK293T_S1’ and ‘u2os_ctrl’, the analysis can be performed by first checking the checkboxes to the right of these two tracks and then click the ‘Compare’ button. The ‘Differential Methylation’ and ‘Differential Expression’ tracks will appear below the section of sample tracks. In this case, we see up-right bars at both site clusters in BRCA1 in the ‘Differential Methylation’ track, indicating hyper-methylation in sample ‘HEK293T_S1’ relative to ‘u2os_ctrl’ at these sites. In contrast, the ‘Differential expression’ track shows that BRCA1 is down-expressed (green block) in ‘HEK293T_S1’ relative to ‘u2os_ctrl’ with a log-fold change of -3.38. These displayed m^6^A peaks in the browser can be exported as a CSV file by clicking the ‘Export Data’ button at the top of the browser. If the user is also interested in gaining a global perspective of the methylation surrounding BRCA1, the user can navigate to the circular ideogram of chromosome 17 (Supplementary Figure S2).

## DISCUSSION

MeT-DB is the first attempt to establish a resource of m^6^A methylation in mammalian transcriptome. MeT-DB corroborates the widespread existence of m^6^A in transcriptome by bringing together predicted m^6^A sites from all existing MeRIP-Seq data sets from different studies. It includes a build-in genome browser to facilitate visualization and comparison of m^6^A methylations in different contexts. As it provides wealth information related to m^6^A, it is a valuable resource for both experimental and computational biologists who aim to improve our understanding of the biological mechanisms and functions of m^6^A.

As m^6^A MeRIP-Seq and other RNA methylation related technologies are being applied to a broader set of species, cell lines, tissues and conditions, the MeT-DB database will be continuously updated and improved. The MeT-DB database currently covers only m^6^A methylation. In the foreseeable future, data from other abundant post-transcriptional RNA methylation, such as 5-methycytocine (m^5^C), will be included as they become available.

## SUPPLEMENTARY DATA

Supplementary Data are available at NAR Online.

## References

[1] Kouzarides T. (2007). Chromatin modifications and their function. Cell.

[2] Thurman R.E., Rynes E., Humbert R., Vierstra J., Maurano M.T., Haugen E., Sheffield N.C., Stergachis A.B., Wang H., Vernot B. (2012). The accessible chromatin landscape of the human genome. Nature.

[3] Dominissini D., Moshitch-Moshkovitz S., Schwartz S., Salmon-Divon M., Ungar L., Osenberg S., Cesarkas K., Jacob-Hirsch J., Amariglio N., Kupiec M. (2012). Topology of the human and mouse m6A RNA methylomes revealed by m6A-seq. Nature.

[4] Meyer K.D., Saletore Y., Zumbo P., Elemento O., Mason C.E., Jaffrey S.R. (2012). Comprehensive analysis of mRNA methylation reveals enrichment in 3’ UTRs and near stop codons. Cell.

[5] Meyer K.D., Jaffrey S.R. (2014). The dynamic epitranscriptome: N6-methyladenosine and gene expression control.

[6] Liu J., Yue Y., Han D., Wang X., Fu Y., Zhang L., Jia G., Yu M., Lu Z., Deng X. (2014). A METTL3-METTL14 complex mediates mammalian nuclear RNA N6-adenosine methylation. Nat. Chem. Biol..

[7] Ping X.L., Sun B.F., Wang L., Xiao W., Yang X., Wang W.J., Adhikari S., Shi Y., Lv Y., Chen Y.S. (2014). Mammalian WTAP is a regulatory subunit of the RNA N6-methyladenosine methyltransferase. Cell Res..

[8] Wang X., Lu Z., Gomez A., Hon G.C., Yue Y., Han D., Fu Y., Parisien M., Dai Q., Jia G. (2013). N6-methyladenosine-dependent regulation of messenger RNA stability.

[9] Jia G., Fu Y., Zhao X., Dai Q., Zheng G., Yang Y., Yi C., Lindahl T., Pan T., Yang Y.-G. (2011). N6-methyladenosine in nuclear RNA is a major substrate of the obesity-associated FTO. Nat. Chem. Biol..

[10] He C. (2010). Grand challenge commentary: RNA epigenetics. Nat. Chem. Biol..

[11] Meng J., Cui X., Rao M.K., Chen Y., Huang Y. (2013). Exome-based analysis for RNA epigenome sequencing data. Bioinformatics.

[12] Fustin J.-M., Doi M., Yamaguchi Y., Hida H., Nishimura S., Yoshida M., Isagawa T., Morioka M.S., Kakeya H., Manabe I. (2013). RNA-methylation-dependent RNA processing controls the speed of the circadian clock. Cell.

[13] Hess M.E., Hess S., Meyer K.D., Verhagen L.A., Koch L., Bronneke H.S., Dietrich M.O., Jordan S.D., Saletore Y., Elemento O. (2013). The fat mass and obesity associated gene (Fto) regulates activity of the dopaminergic midbrain circuitry. Nat. Neurosci..

[14] Wang Y., Li Y., Toth J.I., Petroski M.D., Zhang Z., Zhao J.C. (2014). N6-methyladenosine modification destabilizes developmental regulators in embryonic stem cells. Nat. Cell Biol..

[15] Kim D., Pertea G., Trapnell C., Pimentel H., Kelley R., Salzberg S.L. (2013). TopHat2: accurate alignment of transcriptomes in the presence of insertions, deletions and gene fusions. Genome Biol..

[16] Feng J.X., Liu T., Qin B., Zhang Y., Liu X.S. (2012). Identifying ChIP-seq enrichment using MACS. Nat. Protoc..

[17] Trapnell C., Roberts A., Goff L., Pertea G., Kim D., Kelley D.R., Pimentel H., Salzberg S.L., Rinn J.L., Pachter L. (2012). Differential gene and transcript expression analysis of RNA-seq experiments with TopHat and Cufflinks. Nat. Protoc..

[18] Lewis B.P., Burge C.B., Bartel D.P. (2005). Conserved seed pairing, often flanked by adenosines, indicates that thousands of human genes are microRNA targets. Cell.

[19] Betel D., Koppal A., Agius P., Sander C., Leslie C. (2010). Comprehensive modeling of microRNA targets predicts functional non-conserved and non-canonical sites. Genome Biol..

[20] Li J.-H., Liu S., Zhou H., Qu L.-H., Yang J.-H. (2014). starBase v2. 0: decoding miRNA-ceRNA, miRNA-ncRNA and protein–RNA interaction networks from large-scale CLIP-Seq data. Nucleic Acids Res..

[21] Giulietti M., Piva F., D'Antonio M., D'Onorio De Meo P., Paoletti D., Castrignano T., D'Erchia A.M., Picardi E., Zambelli F., Principato G. (2013). SpliceAid-F: a database of human splicing factors and their RNA-binding sites. Nucleic Acids Res..

[22] da Cunha P.A., de Carlos Back L.K., Sereia A.F., Kubelka C., Ribeiro M.C., Fernandes B.L., de Souza I.R. (2013). Interaction between obesity-related genes, FTO and MC4R, associated to an increase of breast cancer risk. Mol. Biol. Rep..

[23] Li G., Chen Q., Wang L., Ke D., Yuan Z. (2012). Association between FTO gene polymorphism and cancer risk: evidence from 16,277 cases and 31,153 controls. Tumour biology : the journal of the International Society for Oncodevelopmental Biology and Medicine.

[24] Kusinska R., Gorniak P., Pastorczak A., Fendler W., Potemski P., Mlynarski W., Kordek R. (2012). Influence of genomic variation in FTO at 16q12.2, MC4R at 18q22 and NRXN3 at 14q31 genes on breast cancer risk. Mol. Biol. Rep..

[25] Zhao M., Sun J., Zhao Z. (2013). TSGene: a web resource for tumor suppressor genes. Nucleic Acids Res..

[26] Forbes S.A., Bindal N., Bamford S., Cole C., Kok C.Y., Beare D., Jia M., Shepherd R., Leung K., Menzies A. (2011). COSMIC: mining complete cancer genomes in the Catalogue of Somatic Mutations in Cancer. Nucleic Acids Res..

[27] Krzywinski M., Schein J., Birol I., Connors J., Gascoyne R., Horsman D., Jones S.J., Marra M.A. (2009). Circos: an information aesthetic for comparative genomics. Genome Res..

[28] Bailey T.L., Boden M., Buske F.A., Frith M., Grant C.E., Clementi L., Ren J., Li W.W., Noble W.S. (2009). MEME SUITE: tools for motif discovery and searching. Nucleic Acids Res..

